# Physical activity is associated with lower cerebral beta-amyloid and cognitive function benefits from lifetime experience–a study in exceptional aging

**DOI:** 10.1371/journal.pone.0247225

**Published:** 2021-02-19

**Authors:** Valerie Treyer, Rafael S. Meyer, Andreas Buchmann, Giovanni A. G. Crameri, Sandro Studer, Antje Saake, Esmeralda Gruber, Paul G. Unschuld, Roger M. Nitsch, Christoph Hock, Anton F. Gietl

**Affiliations:** 1 Institute for Regenerative Medicine (IREM), University of Zurich, Zurich, Switzerland; 2 Department of Nuclear Medicine, University Hospital of Zurich, University of Zurich, Zurich, Switzerland; 3 Hospital for Psychogeriatric Medicine, Psychiatric University Hospital Zurich, Zurich, Switzerland; 4 Neurimmune, Schlieren-Zurich, Switzerland; Nathan S Kline Institute, UNITED STATES

## Abstract

**Background:**

Exceptional agers (85+ years) are characterized by preserved cognition presumably due to high cognitive reserve. In the current study, we examined whether personality, risk and protective factors for dementia as well as quality of life are associated with core features of Alzheimer’s disease (amyloid-deposition and hippocampal volume) as well as cognition in exceptional aging.

**Methods:**

We studied 49 exceptional agers (average 87.8 years, range 84–94 years), with preserved activities of daily living and absence of dementia. All participants received a detailed clinical and neuropsychological examination. We used established questionnaires to measure lifetime experience, personality, recent physical and cognitive activity as well as quality of life. Cerebral amyloid-deposition was estimated by 18-[F]-Flutemetamol-PET and manual hippocampal volumetry was performed on 3D T1 MRI images.

**Results:**

In this sample of exceptional agers with preserved activities of daily living, we found intact cognitive performance in the subjects with the highest amyloid-load in the brain, but a lower quality of life with respect to autonomy as well as higher neuroticism. Higher self-reported physical activity in the last twelve months went with a lower amyloid load. Higher self-reported leisure-time/ not work-related activity went with better executive functioning at older age.

**Conclusion:**

Even in exceptional aging, high amyloid load may subtly influence personality and quality of life. Our findings support a close relationship between high physical activity and low amyloid-deposition and underscore the importance of extracurricular activities for executive functions. As executive functions are known to be a central resource for everyday functioning in fostering extracurricular activities may be effective in delaying the onset of dementia.

## Introduction

According to the WHO, life expectancy at birth in Europe increased from 72.0 years in 2000 to 77.5 years in 2016, with a healthy life expectancy at birth of 68.4 years. As age is the most important risk factor for dementia, this was paralleled by a rising number of patients with dementia worldwide (50 million of people with dementia in 2019 [1]). Interestingly, recent epidemiological studies showed that age-specific incidence of dementia decreased by 24% from 1990 to 2000 in high-income countries supposedly due to beneficial changes in modifiable risk factors like higher education and reduction of vascular risk factors [2, 3].

Pathologies accumulating with higher age are beta-amyloid deposits and neurofibrillary tangles containing misfolded Tau protein, the typical hallmarks of Alzheimer’s disease. Also cerebrovascular brain lesions t and other additional protein aggregates like TDP43 or alpha-synuclein become increasingly prevalent with age, and could exert a synergistic detrimental impact on the brain [4–7]. Recent developments in brain imaging brought the possibility to visualize certain pathologies. By using positron emission tomography with dedicated radiopharmaceuticals we can measure amyloid- and tau accumulation in the brain [8–13]. Amyloid-pathology accumulates in the brain over decades before first cognitive symptoms of Alzheimer’s disease occur [8, 14]. Over time the decrease in brain tissue especially in mediotemporal regions can be measured as a consequence of disease progression already in a preclinical/presymptomatic stage [15–19]. However, atrophy in the mediotemporal region is not specific to Alzheimer’s disease but occurs in various other neurodegenerative diseases, and up to a certain degree also in normal aging [15, 20, 21]. Volume losses may be associated with cognitive impairment [15, 20–22]. In a population older than 85 years the limbic predominant age-related TDP-43 encephalopathy (LATE) is frequently detectable, but due to lacking in vivo diagnostics for humans it can only be diagnosed post mortem [7].

Populations of exceptional agers offer a unique possibility to identify factors that moderate the link between pathology and cognition [23–27]. Here the concept of cognitive reserve is of high relevance. Cognitive reserve is regarded as the collection of factors that counteract the impact of accumulation of cerebral pathologies and preserve a subject’s cognitive functions [28–31]. To investigate the moderating influence of cognitive reserve, we examined a population of subjects of 85 years and older without evidence for dementia and intact activities of daily living. The central research question was whether established risk- and protective factors for clinical dementia are also associated with biomarkers of Alzheimer’s disease pathology and cognitive functioning in a sample of exceptional agers. Previous studies have shown relationships between dementia incidence and protective factors like cognitive and physical activities [32–36], lifetime experience [20, 37–40], or personality traits [41–44] but findings were equivocal. Higher physical activity was associated with lower brain amyloid uptake in Apolipoprotein E (ApoE) ε4 carriers and in participants with better cognitive performance [45–47], but other studies did not find this relationship (for example, ref [48]). In this study, cognitive functions of older adults (70–89 years old) did not benefit from a two years physical activity program [48]. Another trial showed, that aerobic exercise training increased hippocampal volume compared to a measurable decline in the control group [49].

Positive effects of lifetime experiences (e.g. education, occupational complexity, extracurricular / leisure time activities) have been postulated due to observations, that these factors are related to less hippocampal atrophy [20, 39], higher cognitive reserve in late-life [50] as well as fewer white matter lesions and less amyloid- deposition [51]. Education seems to moderate associations between pathologies and cognition [52] but may not directly contribute to pathology [53]. Cardiovascular risk factors like physical inactivity, unhealthy diets, smoking, obesity and hypertension are modifiable and thus may serve as targets for dementia prevention strategies [54–56]. The impact of these factors on dementia, diabetes and cardiovascular events depend on genetic and other external factors [57–59]. It was repeatedly demonstrated that multifactorial interventions to modify risk factors are useful to prevent neurodegeneration and dementia [60–63].

The concept of exceptional aging provides a framework to understand compensation or protective mechanisms in healthy aging despite the accumulation of various pathologies, while the study of younger currently non-demented cohorts will necessarily include subjects towards pathways of non-successful and successful aging [64–66].

We believe that a thorough investigation of such a population could provide important insights for disease prevention strategies. To add to this picture the design of our study, aims to address important risk factors and their relationship to amyloid deposition and hippocampal volume as biomarkers of brain pathology, and cognition. Risk factors of interest in our study are life experiences, cognitive and physical activity. These were complemented with measures for quality of life and subjective memory complaints as possible subtle consequences of Alzheimer’s pathology as these factors were associated with Alzheimer pathology biomarkers in younger cognitively healthy populations [67–70]. Furthermore we studied personality traits as they could either be a risk factor for Alzheimer’s dementia or directly influenced by Alzheimer’s pathology [41–43, 71].

## Materials and methods

49 older adults (mean age 87.8 year (SD 3.0y)), recruited via advertisement or direct mailing, participated in this study. Parts of this data set have been published previously, comparing a part of the present sample with younger-old subjects with respect to plaque and iron load, entorhinal cortex volume and white matter hyperintensities [72]. The oldest participant was 94 years at baseline and the youngest subject was enrolled 3.5 months before his 85th birthday.

The clinical work-up included assessment of medical history (in particular, prior episodes of brain disease, e.g. ischemia, trauma) and familial history for neurodegenerative diseases, psychiatric, neurological, and internal medicine status. Furthermore ECG and routine laboratory testing, Mini Mental Status Examination (MMSE) [73], Instrumental Activities of Daily Living Scale (IADL) [74] and Hamilton Rating Scale for Depression [75] as well as ApoE genotyping were performed in all patients.

Main inclusion criteria were age of 85 or older and preserved everyday functioning. Main exclusion criteria were clinical dementia as assessed by clinical evaluation or significant neurological, psychiatric or other diseases that may influence cognition or may interfere with the participant’s ability to give informed consent or with compliance at the visits. The study was conducted in accordance with the local law, the Declaration of Helsinki [76] and approved by the ethics committee of the canton Zurich.

### Neuropsychology and domain-specific indices

Standard neuropsychological, psychiatric and neurological assessments were performed. The rationale for test selection was to complement standard dementia tests with tests with a higher cognitive load for each cognitive domain.

After a short informal interview to assess subjective cognitive complaints and factors with possible influence on the test results (e.g., education, alcohol consumption and medication, recent long-distance travel over multiple time zones) as well as characteristics of free language expression, the following neuropsychological tests were administered in the same order: the German version of the CERAD battery [77]; phonematic fluency over three minutes (letters) [78]; the German version of the RAVLT (VLMT; [79]); the Rey-Osterrieth Complex Figure ([80]; including immediate and late recall, evaluated with Taylor’s criteria, [81]); Digit span and Corsi block task (each forward and backward, as in WMS-R; [82]), nonverbal fluency test (5-point test [83]) and Tower of London [84].

All individual test results were z-transformed based on age- and partially years of education corrected clinical norms. The composite scores were generated by averaging all individual transformed test scores relevant for the respective domain. The episodic memory score included the subscores learning, recall and recognition based on VLMT task for learning and recognition, and CERAD’s word learning, VLMT late recall and figures recall from the CERAD battery. The working memory score consisted of Digit span and Corsi block backward. The visuospatial construction score equals the Rey Figure copy, and the naming score equals the short version of the Boston naming test. The executive score included the subscores fluency (verbal and nonverbal fluency tests), non-fluency (colour-word interference and set switching) and error control (based on the sum of errors in three different tests: phonematic fluency, Stroop and Tower of London). The error control scores was a sum of the z-scores and multiplied with (-1) to get higher values for better performances.

### Questionnaire-based scales

For the lifetime experience questionnaire, the same weight scores for the sub-items were used as in an Australian cohort (appendix C of the original publication) [85]. The questionnaire covers three stages of aging, Young Adulthood from 13 to 30 years covering the educational and self-identity finding and first career stage. Mid-life from 30–64 years is characterized by a focus on work-life balance, maintenance of relationships and growing importance of caregiving for children or parents. Late life starts at age of 65 years where many people are retiring and have to reorient their activities beyond work. The questions for each life stage comprise two sets. The first consists of questions on education and work-related activities and the second consisted of general questions about leisure, physical and social activities. We labeled those as ‘extracurricular activities’, as they are not directly related to work or education. Sample questions for these activities are how often participants played or practiced a musical instrument, did artistic pastime, sports or other physical activities, how often they met with friends or family, how often they read, whether they spoke a second language or how far they have travelled across continents.

The WHOQOL-OLD questionnaire in its original German version by the WHO was used to assess quality of life (QOL) in the six domains: sensory abilities, autonomy, past, present and future activities, social participation, death and dying as well as intimacy [86]. The questionnaire consists of 24 questions answered on a 5-point scale. The questions assess QOL over the last two weeks. Sample Questions for the six domains are: “To what extent do impairments to your senses (e.g., hearing, vision, taste, smell, touch) affect your daily life?”, “How much freedom do you have to make your own decisions?” “How much do you feel that you have received the recognition you deserve in life?”, “How satisfied are you with the way you use your time?”, “How scared are you of dying?”, “To what extent do you experience love in your life?”.

To assess current cognitive and physical activities over the last 12 months, a standardized questionnaire was used as described in the original publication [32]. The questionnaire assesses ten cognitive activities like reading, playing games and music, arts and crafting activities as well as social activities were measured. Watching TV for a certain amount of hours did not count for the final cognitive score. In addition the questionnaire covers six physical activities, namely light activities like laundry, vacuuming and light exercises like walking; moderate activities and exercise like gardening or aerobics and strength training as well as information on heavy activities and vigorous exercises like heavy digging, or tennis and jogging. All activities were weighted according frequency of performance of once a month or less (0 points), 2–3 times per month (0.5), 1–2 times per week (1.5), 3–4 times per week (3.5), 5–6 times per week (5.5) and 7 points for daily activities. For cognitive activities a maximum of 70 and for physical activities a maximum of 42 could be reached.

The NEO-FFI was applied to assess the five personality domains, with a particular interest neuroticism as its probable relationship with healthy aging and Alzheimer`s-related pathology [87].

Subjective cognitive complaints were assessed according to Schmand et al.,1996 [70]. This questionnaire consists of 10 items on memory complaints. Example questions are whether other people found the participants forgetful and how often they forgot things, became confused or had concentration problems.

### Imaging based Alzheimer’s diseases biomarkers

All participants received a standard dynamic PET/MR (Signa PET/MR GE Healthcare) scan with approx. 140MBq of 18[F]-Flutemetamol. A BRAVO 3D T1MRI sequence with voxel size 1mm (8-channel coil) was acquired in parallel to calculate cortical beta-amyloid SUVR with bilateral cerebellar grey matter reference for the PET data analysis, which was conducted with the PMOD NeuroTool 3.6 (PMOD Technologies LLC., Switzerland).

To minimize white matter spillover signal and to reduce partial volume effects from cerebrospinal fluid in grey matter regions of interest in the PET data, MRI images were segmented with 3 probability maps. Cortical regions of interest were defined according to Hammer’s maximum probability atlas. Deep nuclei parcellation was performed with NeuroTool (knowledge based, with 20 reference sets). The cut-off for grey matter–white matter segmentation was selected at 50% probability. Automatic outlining of the regions of interest was evaluated by an expert (VT) in each individual scan and corrected manually in PMOD if necessary. Late phase uptake values were calculated as average late frames activity (85 to 100 minutes post injection). The region of interest estimating cortical amyloid-load was defined as the average of cortical Hammer’s atlas defined regions that are overlapping with centiloid regions definition [88] thus not including regions in mediotemporal, occipital, central structures as well as pre- and postcentral gyrus.

A higher-resolution 3D T1 FSPGR (IR600, voxel size 0.5mm) image scanned on a 750W 3T (32-channel coil)-scanner was used for manual outlining of hippocampal volume by an expert (AB).

Volumes were outlined using MRICroN on the coronal slices of the 3D images (with the axial and sagittal views open to verify). The most posterior slice of the anterior hippocampus was outlined as the last (most posterior) slice where the upper part (‘cover’) of the hippocampus goes as far medial as the lower part in an unbroken sheet of grey matter (before the ‘snail’-shape starts; within-structure landmark). Each hippocampus was outlined in an anterior-to-posterior fashion. Fimbria and fornix were excluded. Dark holes were cut out if they were wider than the size of the pen. Volumes in cm^3^ were divided by whole brain volume as derived from SPM 8 VBM (fil.ion.ucl.ac.uk/spm) and results were multiplied by 10’000 for readability. Total hippocampal volume was calculated as averaged sum of the left and right anterior plus posterior hippocampal volumes.

### Statistics

Statistical analysis was done with SPSS 25 (IBM). As this is an exploratory study, all p-values are reported uncorrected. The number of analyses done for each research question is provided in the result sections. SUVR values were used in the analyses as continuous variable or as quartile groups. Median cortical SUVR values were 1.41 (IQR 0.3) (mean 1.56, SD 0.48) and ranges based on quartiles were as follows: Q1 = 1.09–1.29, Q2 = 1.29–1.40, Q3 = 1.40–1.60, Q4 = 1.60–3.2.

Nonparametric testing was performed wherever assumptions for parametric testing (Gaussian distribution as assessed by Kolmogorov-Smirnov test) were not met, which was the case in the majority of variables. In supplemental S1 Table we listed all continuous variables with the results of the Kolmogorov-Smirnov test. To include potential confounders we used partial regression or general linear modelling to explore the effects of potential confounders. The continuous variables were transformed by replacing outliers with cutoff values (standard deviation >3) and rescaled them with a Box-Cox transformation to mean 0 and standard deviation 1 as implemented in SPSS.

Spearman’s tests were used for the primary correlational analyses without adjustment for age, sex and education. Significant correlations were additionally tested with partial correlations with the respective covariates age, sex and education using the Box-Cox transformed continuous variables.

In a similar vein, group comparisons were calculated with Kruskal-Wallis tests (3 or more categories) or Mann-Whitney U tests (2 categories); significant comparisons were explored further with the respective general linear modelling methods including the covariates age, sex and education using the Box-Cox transformed variables.

ApoE 4 Genotype was not included in the analyses due to the low number of ε4 carriers (6) half of which had the combination ε2/ε4.

All tests were performed two-sided.

Power analysis for correlations was calculated using the final sample size N = 49 and type I error of 0.05. With the program G*power the post-hoc power calculation for correlation analysis was used testing against an H0 hypothesis of a rho = 0 [89].

## Results

### Description of study population

**Fig 1:**
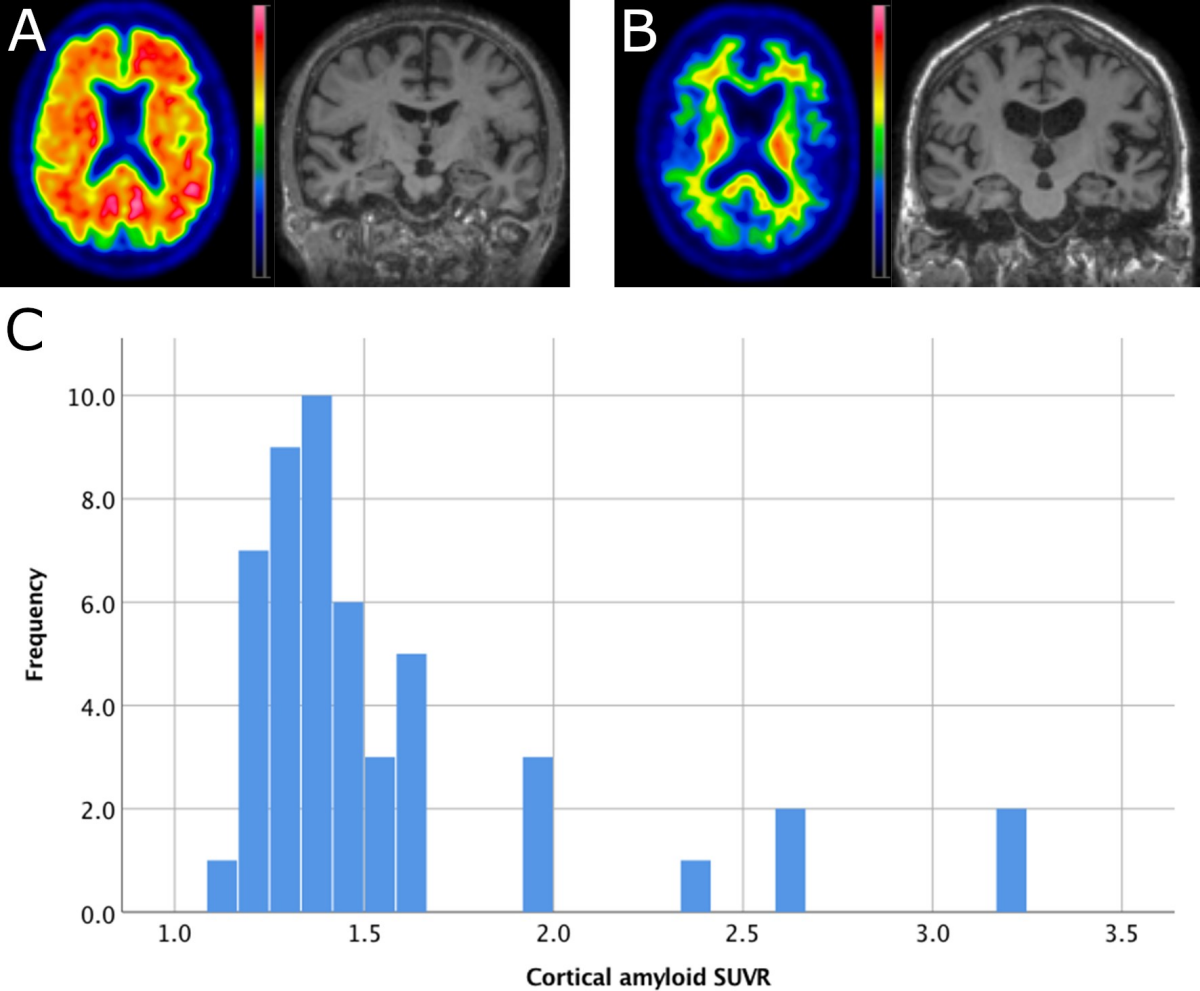
Example Images and distribution of amyloid SUVR. A: Example of a participant with amyloid SUVR = 3.2, age = 86, MMSE = 29 and ApoE = ε3/ε3. The 4mm Gaussian filtered image displays SUVR is scaled from 0–4.5 SUVR. B: example of a participant with amyloid SUVR = 1.2, age = 86, MMSE = 29 and ApoE = ε3/ε3. The SUVR image is scaled from 0–3.5 SUVR. C: histogram of cortical amyloid SUVR (reference region was bilateral cerebellar grey matter).

Table 1 summarizes demographic and clinical parameters of the study population. The IADL includes the actual points of subject scores irrespective whether a task was never performed in lifetime or could not be performed due to other reasons, e.g., physical incapacity. Fig 1 shows a horizontal section of an 18[-F]-Flutemetamol scan indicative of high amyloid load (A) and with low amyloid load (B) as well as the distribution of cortical SUVR values in the study population.

**Table 1:** Overview on study sample.

	Mean (SD) or number (%)
Age	87.8 years (2.99)
sex (female/male)	15 / 34 (30.6% / 69.4%)
Education	14.12 years (2.9)
Education by sex (female/male)	12.87 (2.2) / 14.68 (3.0)
BMI	25.6 (3.2)
Hamilton score	1.35 (2.1)
MMSE	28.35 (1.8)
IADL items	7.33 (1.6)
living on their own alone	26 (53.1%)
living at home with partner	20 (40.8%)
living in dedicated home for older adults	3 (6.1%)
nursing support at home	3 (6.1%)
domestic help/ cleaner used	30 (61.2%)
active driving	29 (59.2%)
ApoE ε3/ε3	34 (69.4%)
ε2/ε3	9 (18.4%)
ε3/ε4	3 (6.1%)
ε2/ε4	3 (6.1%)
**Risk factors and cardiovascular disease**	
self-reported cognitive reduction noted (only 3 confirmed by relatives)	23 (46.9%)
sleeping problems	13 (26.5%)
current smokers (2 only pipes)	3 (6.1%)
never smoked	22 (44.9%)
smoked in the past	24 (49%)
alcohol <80ml	43 (87.8%)
alcohol >80ml	1 (2%)
no alcohol	5 (10.2%)
arterial hypertension	39 (79.6%)
Diabetes	4 (8.2%)
Hypercholesterinaemia	20 (40.8%)
history of myocardial infarction	9 (18.4%)
first grade relatives with dementia	5 (10.2%)

Data is presented as mean and standard deviation or number of cases and percentages of the total study population.

Whole brain volume corrected hippocampal volumes (left plus right side) were on average 9.5 (SD 1.46) with a range of 7.6 to 15.33.

No correlations were detectable between cerebral amyloid-load and hippocampal volume.

### Sex and age effects

Women and men did not differ in age, whole brain volume corrected hippocampal volume and amyloid-load. Compared to men, women had fewer years of education (Mann-Whitney U = 370.5, p = 0.011) and less total lifetime experience in midlife (Mann-Whitney U = 367.5, p = 0.014), especially in job-related categories (Mann-Whitney U = 464, p = 0.001). No differences were seen in the neuropsychological composite score except for episodic memory recall score where women scored better (Mann-Whitney U = 139, p = 0.032).

Neither cerebral amyloid-load nor hippocampal volume correlated with age. In addition, no neuropsychological performance variable correlated with age (note that z-values were partially age-corrected and age range was limited in this study).

### Cognitive performance–no association with imaging biomarkers

MMSE score did not correlate with corrected hippocampal volume nor with amyloid-load. Neuropsychological test performance did not correlate with amyloid-load in any of the cognitive domains.

One significant and negative correlation between cognitive composite scores and hippocampal volume was identified with the recognition score (Spearman’s rho = -0.297, p = 0.042). The partial correlation using transformed variables and with covariates age, sex and education the correlation remained was significant (r = -0.317, p = 0.036, df = 42).

Table 2 lists the values of the composite scores overall and for the groups representing first and fourth quartile of amyloid deposition. None of the differences were significant (Mann-Whitney U: p > 0.25). As some participants did not perform all tasks the number of datasets is indicated next to the composite scores in brackets.

**Table 2:** Neuropsychological assessment results.

Composite Scores for Domains (N completed)	All subjects mean (SD)	Q1 Amyloid load	Q4 Amyloid load	ε2/ε3 or ε3/ε3	ε2/ε4 or ε3/ε4
Working Memory score (48)	-0.30 *(0*.*9)*	**-0.11 *(0*.*9)***	-0.43 *(1*.*0)*	-0.35 (0.8)	**0.11 (1.1)**
Learning score (47)	-0.05 *(0*.*8)*	**-0.02 *(0*.*7)***	-0.15 *(1*.*0)*	-0.10 (0.8)	**0.33 (0.8)**
Recall score (47)	-0.18 *(0*.*9)*	**-0.13 *(0*.*8)***	**-0.07 *(1*.*0)***	-0.30 (0.9)	**0.61 (0.7)**
Recognition score (47)	-0.47 *(1*.*2)*	-0.64 *(1*.*1)*	-0.52 *(1*.*4)*	-0.53 (1.2)	**-0.07 (1.1)**
Executive Functions score (44)	0.07 *(0*.*5)*	0.03 *(0*.*4)*	-0.06 *(0*.*5)*	0.04 (0.5)	**0.30 (0.4)**
Fluencies subscore (46)	-0.01 *(0*.*7)*	-0.08 *(0*.*7)*	-0.19 *(0*.*6)*	-0.02 (0.7)	**0.08 (0.6)**
Not Fluencies subscore (46)	0.21 *(0*.*6)*	**0.30 *(0*.*4)***	0.14 *(0*.*5)*	0.19 (0.6)	**0.39 (0.3)**
Error Control subscore (45)	-1.02 *(1*.*0)*	**-0.80 *(0*.*8)***	-1.50 *(1*.*4)*	-1.07 (1.0)	**-0.67 (0.5)**
Visuo Construction score (47)	-0.14 *(0*.*9)*	**-0.11 *(1*.*3)***	**0.01 *(0*.*8)***	**-0.07 (0.8)**	-0.69 (1.7)
Naming score (48)	0.60 *(1*.*1)*	**0.66 *(1*.*1)***	0.52 *(1*.*4)*	0.50 (1.1)	**1.30 (0.8)**

Z-scores of the composites derived from the neuropsychological examination. Executive functions score is split in three sub scores. The first column lists total average scores with standard deviation in parentheses, the next two columns list the scores of the participants with lowest and highest amyloid load (quartile 1 and quartile 4 of cortical amyloid SUVR). No differences in cognitive functions were observed with respect to amyloid load (*p*>0.25 Mann-Whitney U Test). Also ε4 carrier and non-carriers were grouped in the last two columns. The respective neuropsychological Z-score values are presented. As there were only 6 ε4 carriers the averages need to be interpreted with care and no statistical comparisons were performed due to this imbalance. Z-scores in bold indicate values higher/better than total average of the sample.

We used the subjective memory questionnaire [70] to assess memory complaints in a standardized manner. 19 (38.8%) participants stated no memory complaints, 29 (59%) stated complaints without resulting daily life problems. Only one subject stated memory complaints with resulting problems. The overall test score was low (total score of 2.1; SD 1.5). There was no correlation of the total score with amyloid-load (SUVR), hippocampal volumes or cognitive tests (12 correlations were performed).

### Highest amyloid load is associated with lower experience of autonomy

Our participants had a total quality of life score of 93.9 (ranging from 63–115, SD 11.3) considering the possible scoring range of 24–120 of the WHOQOL-OLD [86].

The average and standard deviation values of the sub scores are listed in Table 3.

**Table 3:** Results of WHOQOL-OLD split by amyloid load and with reference values.

WHOQOL-OLD Facets	Study Sample	Q1 Amyloid load	Q4 Amyloid load
	mean (SD)	mean (SD)	mean (SD)
Sensory Abilities	15.00 (3.27)	16.42 (2.71)	13.83 (4.04)
Autonomy	16.49 (2.39)	16.42 (2.50)	15.25 (2.99)
Past, Present and Future activities	16.02 (2.11)	16.25 (2.22)	15.67 (2.06)
Social Participation	15.39 (2.58)	14.67 (2.31)	14.92 (3.09)
Death and Dying	15.35 (3.69)	16.17 (3.41)	15.17 (3.86)
Intimacy	15.69 (3.30)	15.42 (4.12)	13.92 (3.45)
Total	93.94 (11.31)	95.33 (9.91)	88.75 (14.05)

Results of the WHOQOL-OLD questionnaire. Values of the subgroups with lowest and highest amyloid load (Amyloid quartile 1 and 4) are displayed.

With respect to the relationship with amyloid the QOL aspect autonomy showed a significant difference between the 4 quartiles of amyloid-load (Kruskal-Wallis 7.9, p = 0.047). Visually the highest amyloid SUVR subgroup showed lower values than the other groups. But the post hoc Mann-Whitney U comparison between Q4 and Q1-3 did not show significance (U = 145.5, p = 0.072). A total of 7 comparisons were made.

In Table 3 the QOL values for the first and fourth quartile are displayed. Over all facets, the average value of the participants in the group with highest amyloid load was lower than the values from the group with lowest amyloid load. None of the direct comparisons between subgroups quartile 1 versus quartile 4 reached significance (Mann-Whitney-U p>0.16).

To test for a potential influence of covariates (age, sex and education) a parametric univariate statistics (general linear model) was computed with Box-Cox transformed variables. The corrected model (F_(6,48)_ = 2.43, p = 0.042, partial eta squared = 0.257). The effects of the covariates were not significant (all p˃0.05 and partial eta squared <0.09) but the effect of the fixed factor amyloid-load (amyloid quartiles) was significant (F_(3,48)_ = 3.879, p = 0.016, partial eta squared = 0.217) showing an effect on the dependent variable autonomy.

### Association of amyloid with neuroticism and extraversion scores

The scores measured with the German version of the NeoFFI [87] were 15.3 (SD 6.6) for neuroticism, 25 (SD 5.9) for extraversion, 28 (SD 6.8) for openness for experience, 34.7 (SD 5.8) for agreeableness and 35.2 (SD 6) for conscientiousness.

Scores for neuroticism (Kruskal-Wallis 9.18, p = 0.027) and extraversion (Kruskal-Wallis 15.22, p = 0.002) were significantly different among the quartiles of amyloid-distribution. Visually the quartile with the highest amyloid-load showed reduced extraversion and increased neuroticism compared to Q1-3. Mann-Whitney U post hoc test between Q4 and Q1-3 showed for both significant results (neuroticism Mann-Whitney U = 319.5, p = 0.023; extraversion U = 106, p = 0.007).

**Fig 2:**
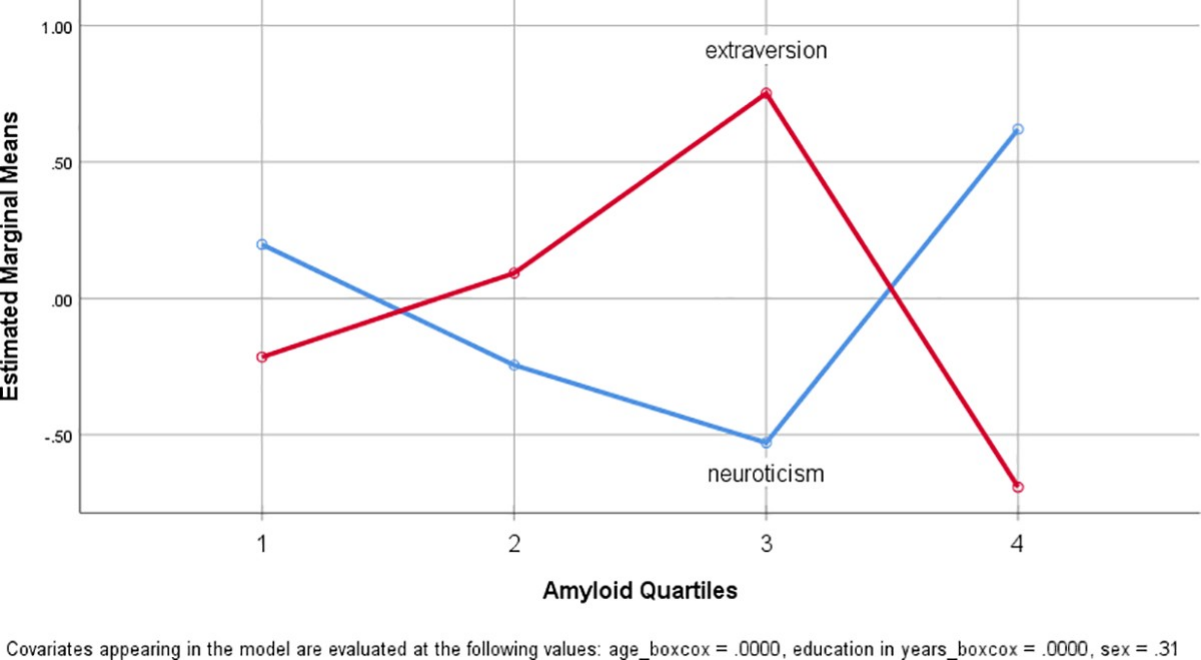
Plot of estimated marginal means of extraversion and neuroticism score against amyloid load. Plot of the estimated marginal means of the two NeoFFI measures as estimated in the repeated measures model with the covariates age, sex and education. Neuroticism against amyloid quartiles is plotted in blue and extraversion in red. Variables were Box-Cox transformed.

To test for the influence of age, sex and education a multivariate analysis for repeated measures (NeoFFI as repeated measure with neuroticism and extraversion) with these covariates and factor amyloid load was performed with Box-Cox transformed variables. No significance for the repeated factor NeoFFI or the covariates (p˃0.6) except for factor amyloid quartiles (F(3,42) = 6.515, p = 0.001, partial eta squared 0.318) (significance remained unaffected by a Greenhouse-Geisser correction as compared to the assumption of sphericity). Fig 2 depicts the marginal means of the two NeoFFI scores against SUVR Quartiles. The comparison between Q1-3 and Q4 amyloid load groups revealed similar results which only amyloid as significant effect with NEOFFI (F(3,44) = 10.33, p = 0.002, partial eta squared 0.190).

Also for between-subjects effects for both personality traits univariate tested the factor amyloid quartiles was significant (neuroticism: F(3,48) = 3.498, p = 0.024, partial eta squared = 0.200; extraversion: F(3,48) = 5.623, p = 0.002, partial eta squared = 0.287). None of the covariates were significant. The corrected model showed a significant effect for extraversion (F(6,48) = 2.940, p = 0.017, partial eta squared 0.296) and showed no significance for neuroticism (F(6,48) = 2.005, p = 0.086, partial eta squared = 0.223).

Participants with lower amyloid-load (Q1-Q3 only) showed a visual but not significant trend of reduced neuroticism scores with higher amyloid-load (rho = -0.233, p = 0.164) and a significant positive correlation with extraversion (rho = 0.402, p = 0.014). SUVR values ranging from Q1-Q3 are normal distributed (Kolmogorov-Smirnov Z = 0.082, p = 0.2).

When performing a partial correlation with control variables age, sex and education and SUVR with all NEOFFI scores extraversion was significantly correlating with SUVR (r = 0.39, p = 0.023, df = 32) while neuroticism was not (r = -0.236, p = 0.178, df = 32). For this test, all variables were transformed within the reduced sample.

Total brain volume-corrected hippocampal volume showed a significant correlation with agreeableness (rho = -0.308, p = 0.031) but none for the other traits. Five comparisons were made for each research question. Performing a partial correlation with age, sex and education as covariates no correlation was significant (p>0.080, with Box-Cox transformed variables).

### Potential protective factors—current physical and cognitive activities

The scores for cognitive activity were ranging from 5–37 and for physical score from 2–35. Values of the score also for subgroups for amyloid load, ApoE genotype and sex are listed in Table 4.

**Table 4:** Results of the cognitive and physical activity questionnaire.

		cognitive activities	physical activities
**Amyloid Quartiles (N)**			
1 (12)	Mean	20.6 (7.2)	11.9 (4.2)
2 (12)	Mean	20.5 (7.9)	10.5 (5.5)
3 (13)	Mean	20.7 (7.5)	10.5 (8.3)
4 (12)	Mean	21.3 (5.4)	9.3 (5.1)
**ApoE Genotype (N)**			
E2/E3 (9)	Mean	23.3 (6.5)	15.9 (8.5)
E2/E4 (3)	Mean	26.0 (5.5)	12.8 (4.3)
E3/E3 (34)	Mean	20.0 (6.9)	9.0 (4.7)
E3/E4 (3)	Mean	17.3 (6.8)	9.7 (1.3)
**sex (N)**			
female (15)	Mean	23.3 (8.0)	11.8 (8.2)
male (34)	Mean	19.7 (6.1)	10.0 (4.7)
**Total (49)**	Mean	20.8 (6.9)	10.6 (5.9)

Table 4: Results of the activity questionnaire with respect to Amyloid load, APOE or sex group. Mean values with standard deviation in parentheses are listed.

**Fig 3:**
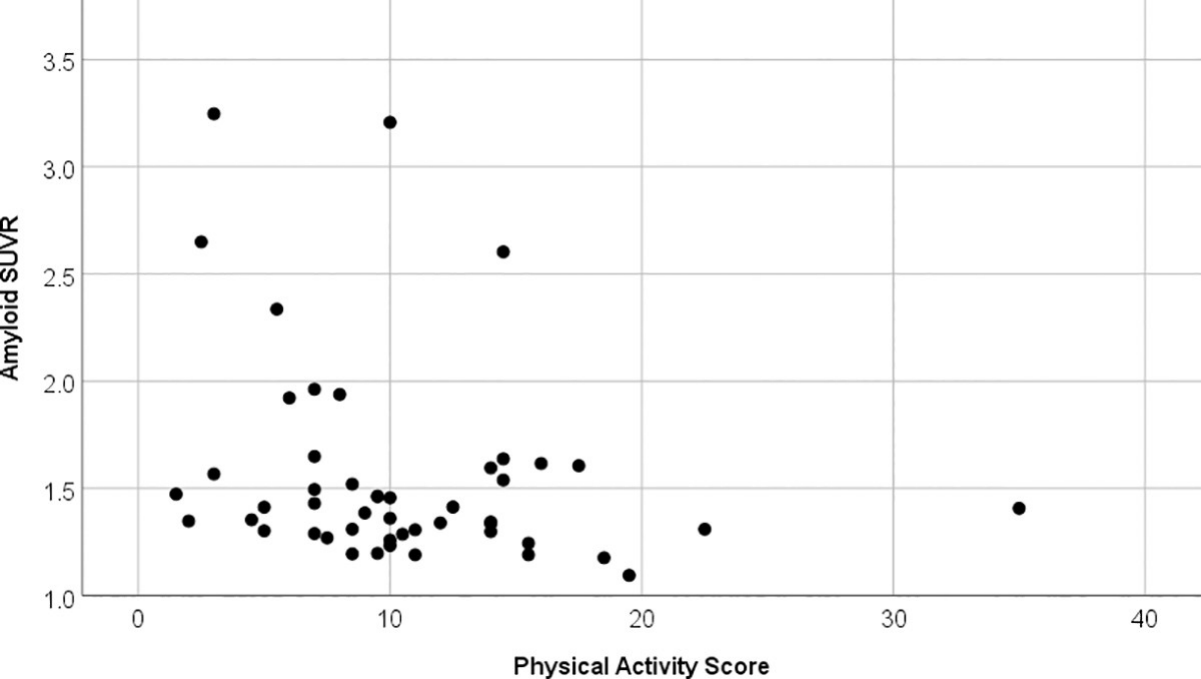
Correlation physical activity score against amyloid SUVR. Scatter plot between physical activity score and amyloid SUVR. The correlation is significant (rho = -0.307, p = 0.032) and indicates that participants with higher physical activity have lower amyloid SUVR.

Physical but not cognitive activity over the last 12 months correlated negatively with cortical amyloid deposition (rho = -0.307, p = 0.032) see also Fig 3. Neither score correlated with corrected hippocampal volume.

Performing a partial regression with age, sex and education as covariates the correlation did remain significant (r = -0.303, p = 0.041, df = 44, performed with transformed variables). Neither age, sex nor education did correlate with a Spearman’s correlation with physical activity score (p>0.27).

### Effects of lifetime experiences

Educational, work-related and extracurricular activities measured with the lifetime experience questionnaire’s (LEQ) [85] were neither associated with cortical amyloid-load nor with hippocampal volume. On average, our sample had a total LEQ of 89.12 (SD 13.1, range 60–120).

Importantly, total lifetime experience correlated with the executive function score (p = 0.014, rho = 0.367) but not with the other assessed cognitive domains. Detailed analysis of the three executive-function subscores revealed that the error control score correlated most strongly with extracurricular and total lifetime experience in all three age stages. The strongest associations were observed for the non-specific subscores (extracurricular activities) but neither for work nor education-related experiences. The partial correlation with covariates age, sex and education showed similar results.

**Fig 4:**
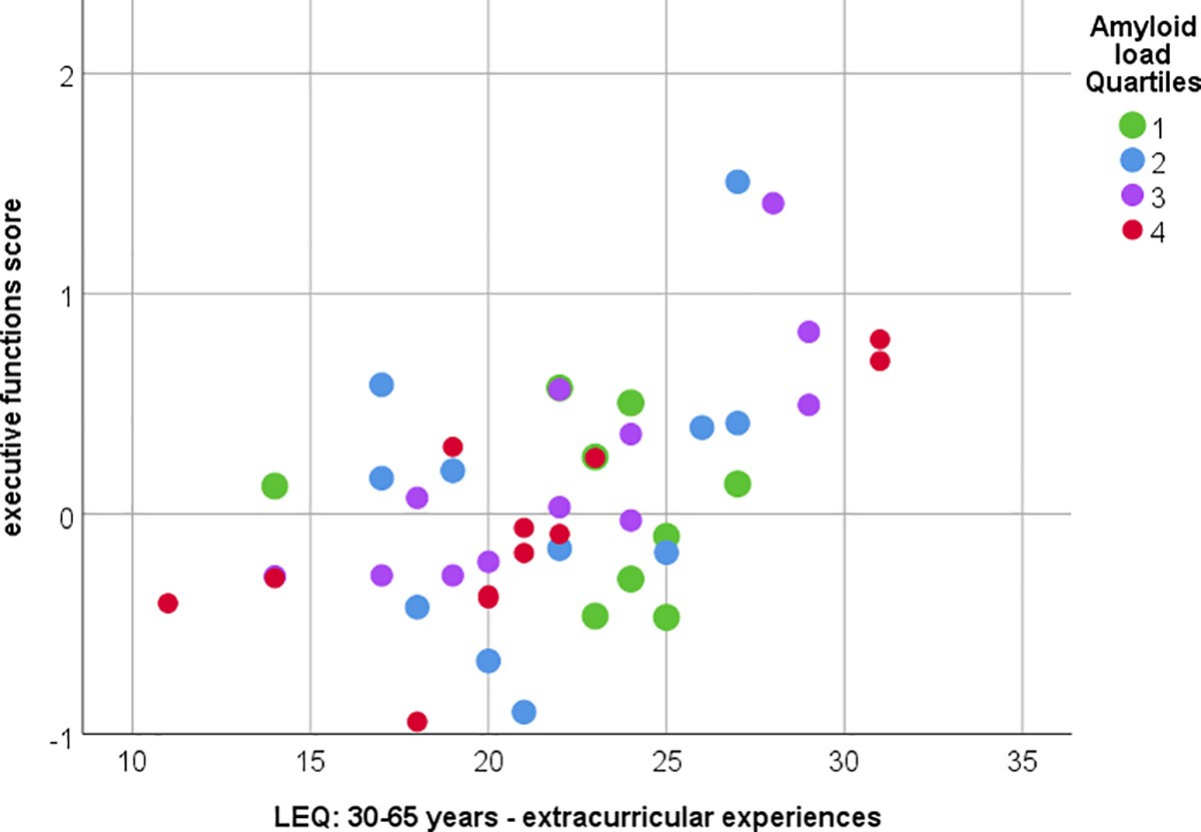
Correlation between midlife extracurricular experiences and executive cognitive functions. Significant correlation between executive function scores and midlife extracurricular experiences (measured with LEQ). Spearman’s rho = 0.513, p<0.001 or Pearson’s partial correlation (with covariates age, sex and education and Box-Cox corrected variables) r = 0.499, p = 0.001. Color indicates amyloid load quartiles (Quartile 1 lowest and Quartile 4 highest amyloid SUVR).

**Fig 5:**
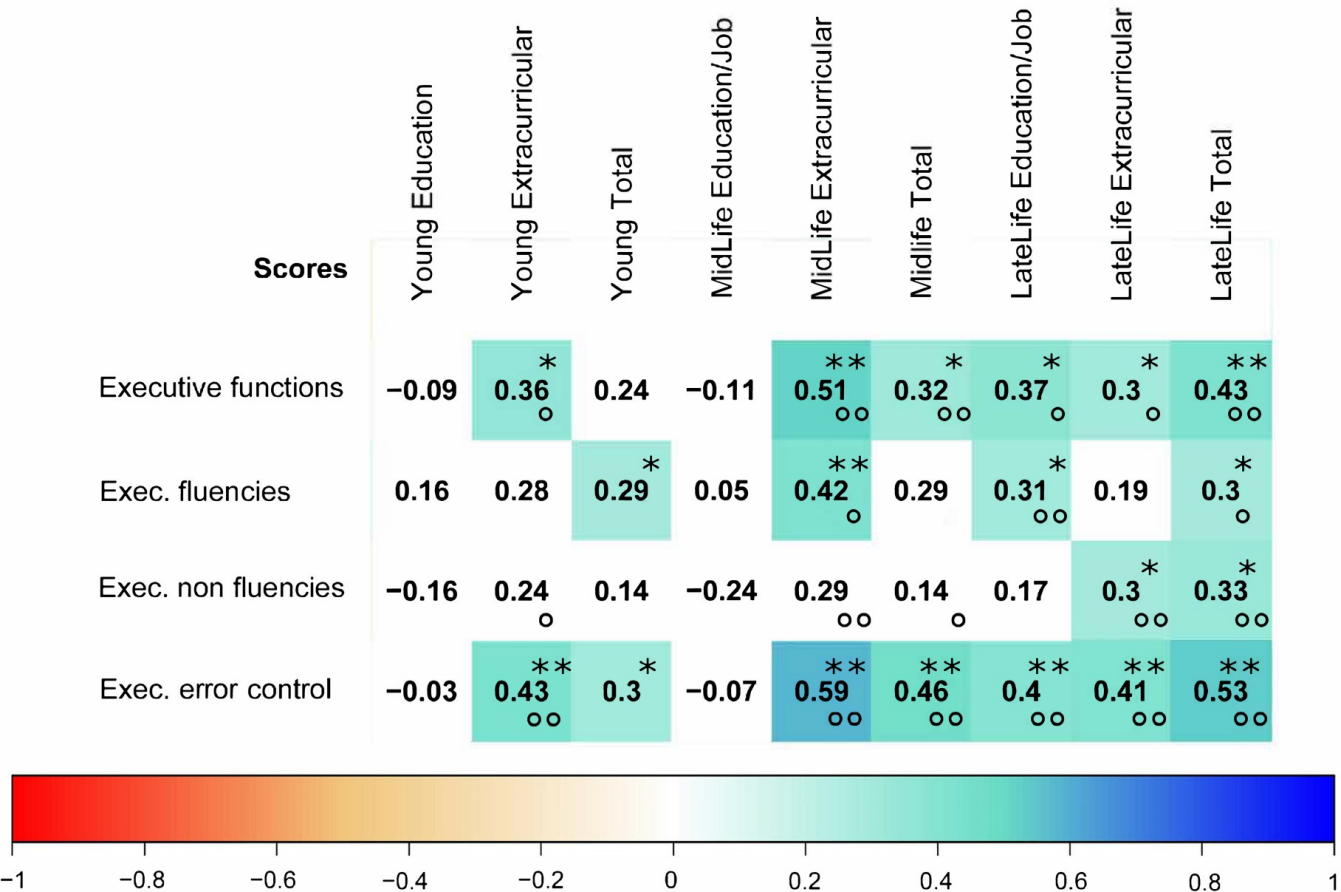
Correlation matrix between lifetime-experiences and executive cognitive functions. Correlation matrix between executive functions scores and Lifetime experience questionnaire scores. Color and numbers indicate the Spearman’s rho value. Color white indicates non-significance (p>0.05). Stars indicating significance level in the Spearman’s correlation and circles significance in the partial correlation with corrected variables (* or ° indicates significance on p = 0.05 level and ** or °° indicates significance on p = 0.01 level).

Fig 4 one correlation between midlife extracurricular experiences and executive cognitive function score is visualized. The correlation matrix between the LEQ items and the executive function scores is visualized in Fig 5 including indications of significance levels for Spearman’s correlation and partial correlation with covariates.

## Discussion

Our selected sample of participants has aged beyond the age of 85 without dementia and with preserved activities of daily living. We have seen that biological markers of Alzheimer’s disease (amyloid and hippocampal volume) and cognitive performance were not related to each other in a way that would reflect the AD typical biomarker scheme where high amyloid and reduced hippocampal volume are related to lower cognitive performance [15, 90].

### High amyloid load- may be associated with lower quality of life and a change in personality

Despite the absence of significant cognitive-amyloid or cognitive-hippocampal volume correlations, we found early signs of amyloid-related effects outside the cognitive domain. We showed the impact of high amyloid load on quality of life with respect to the experience of autonomy and on personality scores.

With respect to a putative impact of personality, our findings indicate that personality traits like extraversion and neuroticism are related to amyloid-load. However, we are examining a sample with relatively low scores for neuroticism and a high quality of life. Our study population showed personality scores comparable to the general population [91], and a 65+ years population in Switzerland [92], with slightly lower neuroticism in our sample. Participants in the highest amyloid-load quartile displayed higher scores for neuroticism and lower scores for extraversion than the three lower amyloid quartiles. This effect was not monotonous along the entire range of amyloid-load.

Increased neuroticism seems to be a preceding signal for dementia [43, 93] as well as for mild cognitive impairment [94, 95]. A study with healthy volunteers (average age 81 years) showed a link between high neuroticism and beta-amyloid deposition in subjects with subjective cognitive complaints [96]. In our smaller and older sample, we did not see the same interaction but subjective memory complaints went with higher neuroticism scores. Various studies have shown changes in personality preceding dementia diagnosis [41, 42, 71, 97, 98]. We therefore suspect a subtle personality change in the high-amyloid subjects rather than a direct biological link as a mechanism to explain the relation.

In addition, we found that subjects with higher amyloid-load had a reduced autonomy score in the quality of life questionnaire. This is consistent with our findings on higher neuroticism and an additional indication that even in subjects with high cognitive reserve, amyloid pathology may become reflected in other, non-cognitive aspects.

### The influence of physical and cognitive activities

We found an association of current physical activity with amyloid deposition, but neither with hippocampal volume nor cognitive measures, in our exceptional agers. The literature on a direct effect of physical activity on amyloid deposition in humans is less compelling than in animal models [99]. Nevertheless, several cross-sectional studies have already shown lower brain amyloidosis in participants with higher physical activity [45–47]. A recent longitudinal study in healthy participants (average 73.4 years) found that more physical activity was associated to slower amyloid related cognitive decline and gray matter volume loss [100]. As we have no longitudinal data on physical activity, we cannot exclude that reduced physical activity could also be a consequence of increased amyloid-deposition that would be consistent with our findings on autonomy and personality.

### Lifetime experience contributes to excecutive functioning

One of the most important protective factors for good cognitive functions at high age is cognitive reserve [30, 90, 101–103]. Our sample displayed a lower amount of cognitive complaints than others [104] which may reflect that our sample consisted of selected cognitively healthy individuals with a high cognitive reserve. Even in our sample, we found lifetime experience and especially extracurricular activities to go alongside with better executive functions in older age. Our results therefore underscore the direct relevance of lifetime experience for executive functioning. This effect did not seem to be mediated by pathology, as increased experience was not correlated with amyloid deposition or hippocampal volume.

In a younger cohort (66–88 years old) a similar relationship between lifestyle and cognition has been shown with a general measure of cognitive ability but without including information about AD biomarkers [50]. A recent study in 330 younger participants without dementia found no effect of midlife cognitive activities on late life amyloid accumulation (i.e., altering pathogenesis) but an effect on late life cognitive performance [105]. Concerning lifetime experience and hippocampal volume a systematic meta-analysis identified an association [106] even though not all studies showed this relationship [32]. However, in a slightly younger population (average 80.8 years) of cognitively intact subjects, a positive correlation between lifetime experience and hippocampal volume was seen, especially with midlife experience score [39].

Especially participants with higher self-paced motivated extracurricular activities profit from better executive functions scores at later life [101, 102, 107–109]. Executive functioning is crucial for mastering everyday life: A recent study demonstrated in 452 MCI patients that executive function is crucial for progression of MCI to dementia [110]. Thus, strengthening lifetime experience and especially extracurricular activities may serve as a central strategy to prevent or increase time to conversion in dementia of all kind, irrespective of pathology.

### Hippocampal volume shows no typical association with memory performance

The negative correlations between hippocampal volume and neuropsychological test performance in our sample were rather unexpected in older adult cohorts (though not unique, see [111]), but have been repeatedly described in younger subjects [112, 113]. In the current cross-sectional analysis, we cannot test if hippocampal atrophy is a risk for further cognitive decline. A recent longitudinal study where non-demented 82+ year old participants were followed up for 7–15 years showed a significant association with memory decline and isolated hippocampal atrophy (in absence of amyloid positive deposition) [114]. In the same study, isolated amyloid positivity (preserved hippocampal volume) was associated with memory and executive decline, while at baseline the effect of amyloid deposition was not significant.

### Methodological considerations

Our sample included only 30.6% women. This is less than in other cohorts and does not reflect the sex proportion in this age group in the general population [115, 116]. Distribution of ApoE genotypes in our sample was similar to other studies in exceptional aging showing low frequencies of the epsilon 4 allele and high frequencies of the epsilon 2 allele. [115–117].

With our sample size and statistical methods, we were only able to detect moderate to strong associations so we cannot rule out subtler effects on AD-biomarkers that would have been uncovered with larger sample sizes, but could still play an important role on an epidemiological level.

We had a power of 83% to detect correlations with effect size of rho = 0.4. For weaker correlations (i.e., rho = 0.3) we only had a power of 56% in our sample.

As this is an exploratory study we chose not to correct our p-values for multiple testing as discussed in detail by others [118]. For all studied comparisons and associations, a scientific rationale existed a priori. In order to capture the specifics of this sample under study we present the different aspects of the study within one publication. We believe that this provides the reader with a more comprehensive picture of the data even if this leads to more comparisons within one publication.

We used state of the art and highly standardized imaging as well as clinical assessment methods to characterize biomarkers, health status and lifestyle factors in this sample. We carefully executed dedicated imaging analysis, taking care of the advanced age of this group and ensuring reliable values.

## Conclusion

We have seen that features of Alzheimer’s pathology occur frequently at higher age in healthy subjects in the absence of dementia. However, the typical association of amyloid with hippocampal volume loss and impaired memory function, which characterizes the pathway to Alzheimer’s disease, is not present in these exceptional agers.

Importantly, our results indicate that even in exceptional aging, high amyloid load may have already subtle effects on personality and quality of life irrespective of effects on cognition. We support the notion of a protective effect of physical activity on amyloid-deposition. In addition, extracurricular lifetime experience plays an important role for later executive cognitive functions irrespective of Alzheimer’s pathology and thus enhancing such experience may serve as a general protective strategy for dementia.

## Supporting information

S1 Table Test for normal distribution with continuous variables of interest. (DOCX)

S2 Table Key parameters grouped in accordance with ApoE genotype. (DOCX)
