# Supplementary material for: Physical activity is associated with lower cerebral beta-amyloid and cognitive function benefits from lifetime experience–a study in exceptional aging
Source: PLoS One. 2021 Feb 19;16(2):e0247225. doi: 10.1371/journal.pone.0247225 (PMC7895362; doi:10.1371/journal.pone.0247225)
Supplement: S1 Table — (DOCX) [file pone.0247225.s001.docx]

**S1 Table: Test for normal distribution with continuous variables of interest**

| **Variable** | **Kolmogorov Smirnov Test statistics** | **Asympt. Sign.** | **Distribution** |
| --- | --- | --- | --- |
| MMSE | 0.259 | 0 | not normal |
| Age | 0.194 | 0 | not normal |
| Years of Education | 0.222 | 0 | not normal |
| WHOQOL-OLD Sensory Abilities | 0.107 | 0.2 | normal |
| WHOQOL-OLD Autonomy | 0.174 | 0.001 | not normal |
| WHOQOL-OLD Past,Present and Future activities | 0.128 | 0.043 | not normal |
| WHOQOL-OLD Social Participation | 0.175 | 0.001 | not normal |
| WHOQOL-OLD Death and Dying | 0.192 | 0 | not normal |
| WHOQOL-OLD Intimacy | 0.17 | 0.001 | not normal |
| WHOQOL-OLD Total | 0.092 | 0.2 | normal |
| LEQ 13-30 education | 0.143 | 0.014 | not normal |
| LEQ 13-30 extracurricular | 0.147 | 0.01 | not normal |
| Total 13-30 | 0.082 | 0.2 | normal |
| LEQ 30-65 job&education | 0.267 | 0 | not normal |
| LEQ 30-65 extracurricular | 0.083 | 0.2 | normal |
| Total 30-65 | 0.123 | 0.062 | normal |
| LEQ 65+ education | 0.164 | 0.002 | not normal |
| LEQ 65+ extracurricular | 0.169 | 0.001 | not normal |
| Total 65+ | 0.103 | 0.2 | normal |
| Total LEQ | 0.136 | 0.024 | not normal |
| NeoFFI N | 0.104 | 0.2 | normal |
| NeoFFI E | 0.144 | 0.013 | not normal |
| NeoFFI O | 0.107 | 0.2 | normal |
| NeoFFI V | 0.084 | 0.2 | normal |
| NeoFFI G | 0.099 | 0.2 | normal |
| Cognitive Activities | 0.081 | 0.2 | normal |
| Physical Activities | 0.13 | 0.037 | not normal |
| Working Memory score | 0.086 | 0.2 | normal |
| Learning score | 0.06 | 0.2 | normal |
| Recall score | 0.111 | 0.196 | normal |
| Recognition score | 0.127 | 0.057 | normal |
| Executive Functions score | 0.083 | 0.2 | normal |
| Fluencies subscore | 0.143 | 0.019 | not normal |
| Not Fluencies subscore | 0.082 | 0.2 | normal |
| Error Control subscore | 0.16 | 0.006 | not normal |
| Visuo Construction score | 0.164 | 0.003 | not normal |
| Naming score | 0.193 | 0 | not normal |
| Amyloid SUVR | 0.26 | 0 | not normal |
| normalized Hippocampus volume | 0.131 | 0.034 | not normal |

S1-Table: Results of the one-sample Kolmogorov-Smirnov test for normal distribution of all variables of interest.
