# Supplementary material for: Physical activity is associated with lower cerebral beta-amyloid and cognitive function benefits from lifetime experience–a study in exceptional aging
Source: PLoS One. 2021 Feb 19;16(2):e0247225. doi: 10.1371/journal.pone.0247225 (PMC7895362; doi:10.1371/journal.pone.0247225)
Supplement: S2 Table — (DOCX) [file pone.0247225.s002.docx]

**S2 Table: Key Parameters grouped in accordance with ApoE genotype**

|  | **ε2/ ε3** | **ε3/ ε3** | **ε2/ ε4** | **ε3/ ε4** |
| --- | --- | --- | --- | --- |
| Cases | 9 (18%) | 34 (69%) | 3 (6%) | 3 (6%) |
| sex – female (100%) | 4 (72%) | 8 (53%) | 1 (7%) | 2 (13%) |
| sex – male (100%) | 5 (15%) | 26 (76%) | 2 (6%) | 1 (3%) |
| MMSE | 28.2 (2.1) | 28.4 (1.4) | 29.3 (1.2) | 26.7 (4.0) |
| Age | 87.5 (3.1) | 87.9 (3.1) | 88.7 (4.2) | 86.3 (0.6) |
| Years of Education | 15.4 (3.4) | 13.9 (2.8) | 15 (2.6) | 12 (1.0) |
| Amyloid SUVR mean(SD) | 1.4 (0.1) | 1.6 (0.5) | 2.1 (0.5) | 1.3 (0.2) |
| Amyloid Quartile 1 | 2 | 8 | 0 | 2 |
| Amyloid Quartile 2 | 4 | 8 | 0 | 0 |
| Amyloid Quartile 3 | 2 | 10 | 0 | 1 |
| Amyloid Quartile 4 | 1 | 8 | 3 | 0 |
| Normalized Hippocampus Volume | 10.0 (1.0) | 9.4 (1.4) | 8.4 (0.7) | 10.4 (2.8) |

S2-Table: Numbers are given as mean and standard deviation values or number of cases and percentages of total where appropriate.
